# Dietary Practices Among Type 2 Diabetes Patients Visiting a Non-communicable Disease (NCD) Clinic in a District of Western India: A Cross-Sectional Study

**DOI:** 10.7759/cureus.52604

**Published:** 2024-01-20

**Authors:** Bela Patel, Sumit Unadkat, Harsh Patel, Mittal Rathod

**Affiliations:** 1 Community Medicine, Swaminarayan Institute of Medical Sciences and Research, Kalol, IND; 2 Community Medicine, Shri M. P. Shah Government Medical College, Jamnagar, IND; 3 Community Medicine, SAL Institute of Medical Sciences, Ahmedabad, IND; 4 Community Medicine, All India Institute of Medical Science, Jammu, Jammu, IND

**Keywords:** observational cross-sectional study, dietary practice, dietary perception, ukddq score, type-2 diabetes

## Abstract

Background and aims: Diabetes is becoming a major public health problem in the country. One of the most important lifestyle modifications necessary for diabetic patients is maintaining healthy dietary choices. These modifications in dietary practices are supposed to be followed lifelong, along with medication, for better glycemic control. Despite understanding the importance of dietary control and physical activity in the management of diabetes, adherence to these practices is poor. This study aimed to assess the dietary practices of type 2 diabetes mellitus (T2DM) patients and various factors that determine adherence to these healthy dietary practices. The secondary objective was to find the perceptions of participants about the role of diet in controlling diabetes and to find the perception-practice gap among study participants.

Methodology: It was a hospital-based cross-sectional study conducted among 450 T2DM patients visiting the non-communicable disease (NCD) clinics of tertiary care hospitals and community health centres (CHCs) of the study district. Dietary practice was assessed using a modified UK Diabetes and Diet Questionnaire (UKDDQ), considering the food patterns in the study area. Statistical tests like chi-square and ordinal logistic regression were applied using Jamovi software for univariate and multivariate analyses.

Results: The healthiest food choices were abstinence from alcohol consumption (100%), avoiding processed meat (92.21%), high-fibre breakfast (70.4%), and daily consumption of vegetables (68.2%). Improper dietary practices were regular sugary drinks (38%) and high-glycemic-index food items (22.4%). The mean (SD) of the composite score was 68.02 (8.7) and the median score (interquartile range (IQR)) was 69 (60-76). Tertile analysis of the composite score revealed that with the increase in age, patients were less likely to be in the intermediate or upper tertile score (β = -0.0219, p = 0.016). Being female (odds ratio (OR) =0.603, CI: 0.395-0.917, p = 0.019) and living in a three-generation family made the patients less likely to be in the upper tertile score.

Conclusion: Nearly half of the participants had an overall healthy score. Dietary practices were healthy among the participants of lower ages, males, and those living in nuclear and joint families. The highest perception-practice gap was seen for fruit and rice consumption.

## Introduction

Diabetes mellitus (DM) refers to a group of common metabolic disorders that share the phenotype of hyperglycemia. Diabetes is becoming a major public health problem in the country, with the number of adults living with diabetes having more than tripled over the past 20 years. The tenth edition of the International Diabetes Federation (IDF) atlas shows that 537 million adults (20-79 years old) are currently living with diabetes. The IDF estimates that there will be 643 million adults with diabetes by 2030 and 784 million by 2045 [1]. According to the World Health Organization (WHO), there is an apparent epidemic of diabetes, which is strongly related to lifestyle and economic change [2].

One of the most important lifestyle modifications necessary for diabetic patients is maintaining healthy dietary choices and eating habits. Apart from that, other critical factors for maintaining optimal blood glucose levels include adequate physical activity, medication compliance, and routine monitoring of blood glucose levels [3]. A healthy dietary practice is among the seven self-care practices recommended by the American Association of Diabetes Educators, which emphasizes that the total diet or overall pattern of food, in quantity and quality, is the most important aspect of a healthy eating style. These modifications in dietary practices are supposed to be followed lifelong, along with medication, for better glycemic control. Diet and eating patterns adopted by individuals are mostly part of the prevailing culture of the region. Despite knowing the facts, individual practice depends on their perception or belief. Sometimes, they also practice contrary to their perception because of some physical, social, or financial constraints.

In India, dietary patterns are highly diverse and heterogeneous, making the process of food pattern evaluation very complex [4]. Despite understanding the importance of dietary control and physical activity in the management of diabetes, adherence to these practices is poor [5]. Modifying this dietary pattern is not easy since it involves personal beliefs, perceptions, and continuous self-motivation. Primary healthcare providers can play an important role in helping patients understand the concept of dietary management and implement it in their daily lives [6]. Studies in the western part of India related to dietary practices among diabetics are lacking. So, the present study was done with the primary objective of assessing the dietary practices of type 2 diabetes mellitus (T2DM) patients and various factors that determine adherence to these healthy dietary practices. The study also aimed to find the perceptions of participants about the role of diet and eating patterns in the control of diabetes and to find the perception-practice gap among the study participants.

## Materials and methods

The Government of India launched a national health programme for the prevention and control of cancer, diabetes, cardiovascular disease, and stroke to strengthen infrastructure, human resource development, health promotion, early diagnosis, management, and referral. Under this programme, non-communicable disease (NCD) clinics are established at the district and community health centre (CHC) levels. We carried out this cross-sectional study among T2DM patients visiting NCD clinics in the study district. The research was done between October 2020 and November 2021.

Sample size

At the 95% confidence level, assuming a 50% prevalence rate of adherence to a diabetic diet, a relative precision of 10%, and a non-response rate of 10%, the sample size was calculated to be 422, which was rounded up to 450 patients. Of the 450 patients, half (225 patients) were enrolled consecutively in the NCD clinic of Shri M. P. Shah Government Medical College, a tertiary care hospital in Jamnagar, Gujarat, India. Seventy-five consecutive patients were enrolled in each of the three NCD clinics of randomly selected CHCs in the Jamnagar district. The inclusion criteria were patients with T2DM of any age, diagnosed for at least one year, and giving consent to participate in the study. The exclusion criteria were patients recently diagnosed within one year and those who did not want to participate in the study. A trained interviewer conducted direct patient interviews and collected necessary information in a pre-designed, pre-tested, and semi-structured questionnaire.

Dietary details

Assessment of dietary practice was done by using an interviewer-administered diet assessment tool that was developed based on the UK Diabetes and Diet Questionnaire (UKDDQ). Food items in the dietary assessment questionnaire were according to the prevailing pattern of food consumption in the study area. The questionnaire has 23 items, of which 22 are directly related to diet patterns. Answers to each question were noted as the frequency of the consumption of food items or dietary practices per week or per day, and a code was given from A to F according to the scheme of score. Codes A and B were categorised as healthy choices, codes C and D as less healthy choices, and codes E and F as unhealthy dietary choices. Codes A to F were further allotted scores like A = 5, B = 4, C = 3, D = 2, E = 1, and F = 0 for analysis purposes. The questionnaire was pretested for clarity, and required changes were made in the framing of the questions.

Perception about the role of diet

Usually, diabetes patients are counselled for necessary dietary modifications either by physicians, dieticians, or primary healthcare workers. There are other sources of diet-related information, like print or electronic media, social networks, friends, and relatives. Participants were asked semi-structured questions to know their perceptions and beliefs about the role of diet in diabetes management. Perception was not measured with any tool.

Statistical analysis

Data were entered in Microsoft Excel 2019 (Microsoft Corp., Redmond, WA) and analysed in Jamovi software version 2.3.21 (The jamovi project (2023). jamovi (Version 2.3) (Computer Software). Retrieved from https://www.jamovi.org). Univariate analysis was done to describe the independent variables (age, gender, area of residence, literacy, and type of family) and presented as a number and percentage. The dependent variable, i.e., the composite score of the dietary assessment tool, was described using the median and interquartile range (IQR). Using tertile analysis, the composite dietary assessment score was categorised as upper tertile, intermediate tertile, and lower tertile. A chi-square test was used to test the significant association between the independent and dependent variables. Independent variables with p <0.2 in univariate analysis were included in the ordinal logistic regression model to control confounding. All the tests were two-tailed, and statistical significance was set at the probability value p <0.05.

Ethical consideration

The study was approved by the Institutional Ethical Committee of M. P. Shah Government Medical College, Jamnagar, Gujarat, with reference number IEC/Certi/77/03/2020. The patient's rights to participate in the study were safeguarded. Informed consent was obtained from all participants.

## Results

Of the total 450 participants, 312 (69.3%) belonged to urban areas, 262 (58.22%) were male, and 150 (33.3%) were 65 years of age or older. The mean age of the participants was 58.98 ± 11.69 years. The majority, 405 (90%), were married, and 213 (47.3%) belonged to the upper or upper-middle socioeconomic class. More than three-fourths (346, 76.88%) were literate. Three hundred and ten participants (68.89%) belonged to joint or three-generation families.

Among the healthy food choices were abstinence from alcohol consumption (100%), avoiding processed meat (415, 92.21%), high-fibre breakfast (317, 70.4%), daily consumption of vegetables (307, 68.2%), and high-fibre bread (240, 53.3%). Salads and fruits were consumed daily by only 19.10% and 11.8% of participants, respectively. Daily consumption of glucose-lowering food items like fish or other omega-3-rich foods was reported by only 55 (12.2%) participants. The majority of the participants avoided sweets or desserts. Among the improper choices of food items, 38% reported having regular sugary drinks, followed by 22.4% reported having high glycaemic index food items, and 48.9% reported having butter, cheese, or ghee in their diet daily. On enquiring about regularity and frequency of meals, half of the participants were having regular meals daily (52.2%), breakfast within two hours of waking up (38.9%), and almost all (98.2%) were avoiding eating in restaurants frequently. But 103 (22.9%) participants reported having the habit of eating sweet biscuits, chocolates, or cake in between meals (Table 1).

**Table 1:** Dietary assessment as per the frequency of intake of food items

Consumption of food items	Healthy	Less healthy	Unhealthy
Fresh green leafy vegetables	307 (68.2%)	143 (31.8%)	0 (00.0%)
Salads	86 (19.1%)	208 (46.2%)	156 (34.7%)
Fruits	53 (11.8%)	168 (37.3%)	229 (50.9%)
Fish, walnuts, flaxseed, or any other omega-3-containing food	55 (12.2%)	51 (11.3%)	344 (76.4%)
Glucose-lowering fibre	56 (12.4%)	175 (38.9%)	219 (48.7%)
High fibre bread	240 (53.3%)	210 (46.7%)	0 (00.0%)
Bowl of whole-grain breakfast cereals or bran with high-fibre content	317 (70.4%)	70 (15.6%)	63 (14.0%)
Pulses	92 (20.4%)	251 (55.8%)	107 (23.8%)
Sugary drinks	207 (46.0%)	72 (16.0%)	171 (38.0%)
High glycemic index food	55 (12.2%)	294 (65.3%)	101 (22.4%)
Packaged savoury foods	346 (76.9%)	69 (15.3%)	35 (07.8%)
Packaged savoury pastry	257 (57.1%)	160 (35.6%)	33 (07.3%)
Dessert/ sweets with meals	400 (88.9%)	50 (11.1%)	0 (00.0%)
Serving of white rice	87 (19.3%)	207 (46.0%)	156 (34.7%)
Butter, cheese, ghee, etc.	144 (32.0%)	86 (19.1%)	220 (48.9%)
Processed meat	415 (92.2%)	35 (07.8%)	0 (00.0%)
Alcohol	450 (100.0%)	0 (00.0%)	0 (00.0%)
Milk	91 (20.2%)	230 (51.1%)	129 (28.7%)
Regular meals in a day	235 (52.2%)	91 (20.2%)	124 (27.6%)
Breakfast within two hours of waking up	175 (38.9%)	95 (21.1%)	180 (40.0%)
Cake, sweet biscuits, cookies, or chocolates between the meals	337 (74.9%)	103 (22.9%)	10 (02.2%)
Fast foods and fried items	442 (98.2%)	8 (01.8%)	0 (00.0%)
Overall diet assessment	49.0%	28.0%	23.0%

The composite dietary assessment score is mentioned in Table 2.

**Table 2:** Composite dietary assessment score and tertile group IQR: interquartile range

Composite score	
Mean (SD)	68.02 (±8.7)
Median (IQR)	69 (60-76)
Tertile groups of the composite score	
Lower tertile (composite score <65)	143 (25.8%)
Intermediate (composite score 65-73)	156 (27.3%)
Upper tertile (composite score >=74)	151 (46.9%)

The mean (SD) of the composite score was 68.02 (8.7) and the median (IQR) was 69 (60-76). Study subjects were categorised into three groups based on tertiles of composite score, i.e., lower score (<65), intermediate score (66-73), and high score (≥74) (Table 2).

Univariate analysis revealed that age group, gender, literacy status, socioeconomic class, and type of family were significantly associated with the tertile group of dietary assessment scores. The proportion of patients aged 45 years and younger was higher in the upper tertile group (36.7%) compared to older patients aged 65 years or older (20.0%). The proportion of patients in the upper tertile score was significantly higher among males (38.2%), literates (35.0%), and those who belonged to joint families (55.9%) compared to females (27.1%), illiterates (28.8%), and those from nuclear (37.9%) or three-generation families (18.1%), respectively. The proportion of patients in the upper tertile score group was highest among socioeconomic class I (50.6%) and lowest in socioeconomic class III (12.0%). Though the percentage of patients from urban areas in the upper tertile score was higher (35.9%) compared to rural patients (28.3%), the difference was not statistically significant (Table 3).

**Table 3:** Association between sociodemographic variables and tertile groups Data have been presented as N (%); the chi-square test has been applied to test significance; p<0.05 is considered significant

Variables	Diet score tertiles			n	p-value
Age group	Lower	Intermediate	Upper		
45 years and less	11 (22.5 %)	20 (40.8 %)	18 (36.7 %)	49	<0.001
46 to 64 years	65 (25.9 %)	83 (33.1 %)	103 (41.0 %)	251	
65 years and more	67 (44.7 %)	53 (35.3 %)	30 (20.0 %)	150	
Gender					
Male	87 (33.2 %)	75 (28.6 %)	100 (38.2 %)	262	0.004
Female	56 (29.8 %)	81 (43.1 %)	51 (27.1 %)	188	
Area of residence					
Urban	102 (32.7 %)	98 (31.4 %)	112 (35.9 %)	312	0.08
Rural	41 (29.7 %)	58 (42.0 %)	39 (28.3 %)	138	
Education					
Illiterate	25 (24.0 %)	49 (47.1 %)	30 (28.9 %)	104	0.009
Literate	118 (34.1 %)	107 (30.9 %)	121 (35.0 %)	346	
Socioeconomic status (SES)					
I	29 (35.8 %)	11 (13.6 %)	41 (50.6 %)	81	<0.001
II	52 (39.4 %)	21 (15.9 %)	59 (44.7 %)	132	
III	24 (32.0 %)	42 (56.0 %)	9 (12.0 %)	75	
IV	30 (23.2 %)	65 (50.4 %)	34 (26.4 %)	129	
V	8 (24.2 %)	17 (51.5 %)	8 (24.2 %)	33	
Type of family					
Nuclear	34 (24.2 %)	53 (37.9 %)	53 (37.9 %)	140	<0.001
Joint	22 (19.8 %)	27 (24.3 %)	62 (55.9 %)	111	
Three-generation family	87 (43.7 %)	76 (38.2 %)	36 (18.1 %)	199	

All the independent variables were entered into the ordinal logistic regression model to control the confounders. The overall model was found to be significant (chi-square value = 63.6, df = 9, p<0.001). Age, gender, and type of family were found to be independent variables in predicting the outcome variable, i.e., the intermediate or upper tertile score. With every one-unit increase in age, patients were less likely to be in the intermediate or upper tertiles (β = -0.0219, p = 0.016). Females had 40% fewer chances of being in the intermediate or upper tertile score group as compared to males (odds ratio (OR) = 0.603, CI: 0.395-0.917, p = 0.019). Patients from a joint or nuclear family were 4.287 and 1.801 times more likely to have an intermediate or upper tertile score compared to those from the three-generation family (OR = 4.287, CI: 2.669-6.957, p<0.001, and OR = 1.801, CI: 1.103-2.947, p = 0.019, respectively, for joint and nuclear families). In comparison to social class I patients, all the other socioeconomic group patients were less likely to be in the intermediate/upper tertile score group. But it was significant for social class III (54.3% less likely, OR=0.447, CI: 0.229-0.861, p=0.017) and social class IV (50.3% less likely, OR=0.497, CI: 0.259-0.945, p=0.034) only. Literacy status was not found to be an independent predictor of improving the tertile dietary assessment score (Table 4).

**Table 4:** Ordinal logistic regression to predict independent variables for the dietary assessment score The dependent variable 'tertile' has the following order: lower, intermediate, upper

Model coefficients							
Predictor	Estimate	Z	p	Odds ratio	95% CI		
					Lower	Upper	
Age	-0.0219	-2.41	0.016	0.978	0.961	0.996	
Gender							
Female – Male	-0.5062	-2.35	0.019	0.603	0.395	0.917	
Education							
Literate – Illiterate	-0.4022	-1.52	0.129	0.669	0.397	1.124	
Socioeconomic status							
II – I	-0.4892	-1.57	0.115	0.613	0.332	1.123	
III – I	-0.8062	-2.39	0.017	0.447	0.229	0.861	
IV – I	-0.6987	-2.12	0.034	0.497	0.259	0.945	
V – I	-0.6076	-1.42	0.155	0.545	0.235	1.255	
Type of family							
Joint – Three-generation	1.4555	5.96	< 0.001	4.287	2.669	6.957	
Nuclear – Three-generation	0.5882	2.35	0.019	1.801	1.103	2.947	

**Figure 1:**
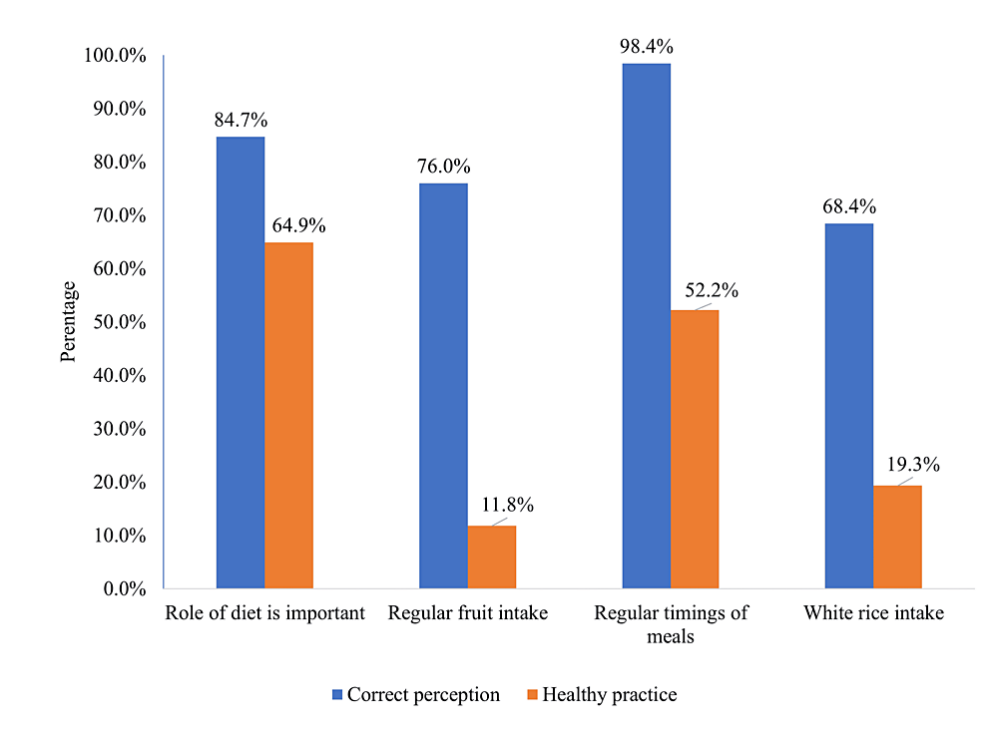
Perception and practices of diet among diabetic patients

When asked about their perceptions about the role of diet, the majority of the participants (381, 84.7%) believed that dietary modification was important for the control of the disease. Three-fourths of the participants (342, 76%) opined that they should consume fruits regularly. Three hundred thirty-three participants (74%) believed that they should reduce the portion size of the food they consume. More than two-thirds (308, 68.4%) believed that they should avoid consuming white rice frequently. Almost all the patients (443, 98.4%) knew that they should maintain regular timing of their meals. When some of these beliefs were compared with their dietary patterns, a perception-practice gap was found. The perception-practice gap was greater for fruit intake, followed by avoiding white rice (Figure 1).

## Discussion

The present study investigates the adherence to healthy dietary practices and perceptions of the role of diet among T2DM patients who are visiting NCD clinics in the study district using a validated measure of the derivative of UKDDQ, a dietary assessment tool. Dietary scores were categorised as healthy at a higher score and unhealthy at a lower score. Our findings illustrate that 46.9% of the participants had a healthy diet based on the composite UKDDQ score. The healthiest dietary choices were found for abstinence from alcohol, not eating fast food, and processed meat, while the least healthy dietary practices were found to be not eating fruits and omega-3-containing foods regularly and having high glycemic index foods. Alcohol is banned in the study area, which could be the reason for the healthy score related to alcohol intake. This finding is supported by the study of Aamir et al., in which only 1.5% of study participants were consuming alcohol due to the complete alcohol ban in the state of Bihar [4]. The majority of the study participants were Hindu by religion and mostly followed a vegetarian diet, which could be the reason for the healthy dietary score related to meat consumption in our study. A 17-year follow-up study conducted by the Adventist Society reported a 74% increase in the incidence of diabetes due to a meat-based diet [7]. Another study in a Buddhist society found that adherence to a vegetarian diet was associated with a 35% lower risk of developing diabetes [8]. In addition, the Rotterdam study showed that a vegetarian-based diet adapted by Satija et al. reduced the risk of diabetes (~18%) and pre-diabetes (~11%) after a follow-up of four to seven years [9]. Fast food consumption had been reported by very few participants in the present study (01.8%), which could be explained by the fact that almost half of the patients were more than 60 years of age and would be avoiding fast food. Other studies have shown that the prudent dietary pattern was associated with a modestly lower risk for T2DM, while the Western dietary pattern was associated with an increased risk for T2DM [10,11]. So, healthy scores of these dietary factors could be explained because of the cultural and sociodemographic characteristics of the study subjects.

The fact that white rice is a staple food in Gujarat may be the reason why our study participants' dietary scores for rice consumption were determined to be unhealthy. The fact is supported by a study done by Bhavadharini et al. on white rice intake and incident diabetes in 21 countries, which found that the highest median (IQR) consumption of white rice was seen in South Asia (including India) at 630 (range: 103-952) g/day. The subgroup analysis by regions showed that the association was most pronounced in South Asia (hazard ratio (HR) = 1.61; 95% CI 1.13-2.30; P for trend = 0.02), followed by the rest of the world [12].

In our study, salad and fruits were consumed daily by only 19.10% and 11.8% of participants, respectively. On the other hand, in research conducted in London by Emadian et al., 71.4% and 68.3% of participants reported daily consumption of fruits and vegetables, respectively [13]. This finding is supported by Hall et al. in their study on global variability in vegetable and fruit consumption, which reported 74% low consumption in India [14]. A meta-analysis on fruit and vegetable consumption and the risk of T2DM by Halvorsen et al. found that there were significant 8%-12% reductions in risk with a fruit intake between 100-500 g/day and a 12%-14% reduction in risk with a vegetable intake between 200-400 g/day [15]. Consumption of pulses in our study was found to be appropriate only in one-fifth of the patients. A systematic review and meta-analysis reported that long-term intake of pulses resulted in a significant reduction of fasting blood glucose in adults with T2DM, as estimated from data from 10 randomized controlled trials (RCTs) (effect size (ES) = 0.54; 95% CI - 0.83- 0.24; p ≤ 0.005; I2 = 78%; PI  - 1.44, 0.37) [16].

A higher percentage (87.8%) of patients showed a habit of taking high to moderate glycaemic index items, i.e., rice, potatoes, etc., in the present study, which is supposed to have a higher risk of uncontrolled T2DM and its complications. Good control of glycaemia can reduce the risk of diabetes complications in the short, medium, and long term [17]. Many food items necessary to meet one's daily needs cannot be purchased by those who are struggling financially. As a result, individuals will be compelled to eat a limited number of certain meals and be subjected to inadequate self-dietary control. These results supported the existing evidence from the study of Bhupathiraju et al., who evaluated the T2DM risk with food patterns based on glycemic index [18]. According to the recommendations of the American Diabetes Association (ADA), to maintain a healthy weight and achieve optimal levels of glycosylated haemoglobin (HbA1c), blood pressure, and lipid profile, nutritional therapy for adults with diabetes should emphasise promoting healthy eating patterns based on key nutrients that are varied, selected, and integrated with the right amount. To achieve this, the ADA emphasises that cultural preferences should be considered, as well as the areas where patients live, access to recommended foods, and a willingness to change. It refers to maintaining the pleasure of eating and providing the necessary tools to empower patients to establish healthy eating patterns themselves, rather than talking about unique foods or micro/macronutrients [19].

Insufficient knowledge about omega-3 is the reason behind the unhealthy nature of omega-3 intake (walnuts and fish). We found that as many as 40% of participants were not having their breakfast within two hours after waking, while one in five participants (21.1%) rarely had breakfast within two hours of waking up. The reason behind this finding could be that half of the participants were women, and the majority of them were homemakers. Once again, cultural practices and beliefs play a role in such practices, such as working late in the morning [4,20]. In our study, 38% of participants reported an unhealthy intake of sugar-sweetened drinks, compared to 69.8% of participants in the Emadian et al. study [13].

The proportion of patients in the upper tertile score was significantly higher among males (38.2%) compared to females (27.1%). A similar result was found by Ntaate where males (78%) had good dietary practices [21]. Although not statistically significant, the proportion of patients in the upper tertile score was higher among those residing in urban areas compared to those residing in rural areas. A similar finding was observed by El-Khawaga et al., who found that 89.6% of urban participants had healthy eating habits [22]. Furthermore, Ayele et al. also found in their study that patients living in a rural area were 2.4 times less likely to adhere to their dietary recommendations for diabetes (adjusted odds ratio (AOR) = 2.423 (95%CI 2.132-4.067) [23]. Such observations could be explained by the possibility that urban patients may have better access to and knowledge about dietary recommendations for T2DM. The proportion of participants in the upper tertile score was significantly higher among literates in our study. Similarly, El-Khawaja et al. and Ayele et al. observed that individuals with elementary educational status were around four times more likely to perform self-care (including dietary care) than those individuals who are unable to read and write (OR=3.9, 95%CI (1.23-12.18)) [22, 23]. While Emadian et al. found no significant differences in the mean UKDDQ score due to differences in levels of education, [13] we found that patients from nuclear or joint families were more likely to have an intermediate or upper tertile score. Horikawa et al. reported that energy intake was significantly higher in diabetic patients who ate at least once a month with their families than in those who did not. [24] In addition, Jeong et al. showed that elderly females had greater total energy as a result of the frequency of having meals with their families [25]. Moreover, in a typical Indian three-generation family or joint family, the traditional dining style is to sit on a mat together and share a meal with the family from the common food pot, a custom that may have led to excessive dietary intake [26]. As age increases, patients are less likely to be in the intermediate or upper tertiles. This finding is in line with the Worku et al. study, where this proportion was lower at 134 (64.73%) in the 60-year-old or older age group compared to <60 years (75.51%) [27].

Most of the participants had the right perceptions about the diabetic diet in the present study. A nearly similar result was found in studies by Gudlavalleti et al. and Bano et al., where 87.7% of participants believed DM was not caused by high sugar intake earlier in life and 73.2% of the participants believed that diet was important in controlling diabetes, respectively [28,29]. Vegetables and fruits are rich in antioxidants, which have been associated with a decreased risk of T2DM [29,30]. A meta-analysis of prospective studies found an association between vegetable and fruit intake and a lower risk of T2DM [30-32]. Since fruits are typically expensive and many of the participants were from lower socioeconomic classes, it is possible that they were unable to purchase them, which may have contributed to the large difference between perceptions and practices regarding fruit consumption.

This cross-sectional study has several limitations to mention. First, there are higher chances of recall bias while answering questions related to dietary practice in the last seven days. The findings of the present study may not be generalised, as patients were selected from the NCD clinic and not randomly chosen.

## Conclusions

Nearly half of the participants had an overall healthy score for dietary practice. The healthiest dietary choices were no alcohol intake, restricted fast food, and processed meat consumption, while the least healthy dietary practices were infrequent intake of fruits, omega-3-containing foods, and intake of high-glycemic-index foods. The highest perception-practice gap was seen in fruit and rice intake among the participants. Dietary practices were healthy among the participants of lower ages, males, and those residing in nuclear and joint families. Observations of the study recommend that dieticians at NCD clinics and community health officers (CHOs) at health and wellness centres should regularly arrange awareness programmes for diabetic patients, which will motivate them to adhere to healthy dietary practices.
